# Fabrication of optimized skin biomimics for improved interfacial retention of cosmetic emulsions

**DOI:** 10.1098/rsif.2018.0332

**Published:** 2018-06-27

**Authors:** Georgios Gkotsis, Jonathan James Stanley Rickard, Anju Brooker, Serafim Bakalis, Liam M. Grover, Pola Goldberg Oppenheimer

**Affiliations:** 1 School of Chemical Engineering, College of Engineering and Physical Sciences, University of Birmingham, Birmingham B15 2TT, UK; 2 Procter and Gamble, Newcastle Innovation Centre, Whitley Road, Newcastle upon Tyne NE12 9TS, UK; 3 Department of Chemical and Environmental Engineering, University of Nottingham, Nottingham NG7 2RD, UK; 4 Department of Physics, Cavendish Laboratory, University of Cambridge, JJ Thomson Avenue, Cambridge CB3 0HE, UK

**Keywords:** skin biomimic, cosmetic emulsions, interfacial retention

## Abstract

Retention of hydrophobic active agents on human skin following the use of skin-care formulations is an important indication of the performance of the deposited product. We have developed a novel system which replicates the interaction between human skin and a cosmetic emulsion to systematically establish and characterize the key parameters driving the retention process at the interface. This included a comprehensive study of the skin's biology and physical properties which influenced the process, the fabrication of advanced, improved skin biomimics, the formulation of a cosmetic model-system emulsion, comprising a hydrophobic active agent i.e. petrolatum, commonly used in cosmetic products, the development of a dedicated and highly consistent deposition rig with a corresponding cleaning set-up and the systematic characterization of retention processes on the developed mimics. This study further explores the interplay of petrolatum with skin biomimics and studies the mechanisms that give rise to improved interfacial retention. Petrolatum has been found to create an occlusive layer on the skin mimic, displaying high coverage from emulsion formulations. The large particle size emulsions yielded improved retention on the developed skin biomimics due to the microstructure of the emulsion and the counter effect of the surfactant.

## 1. Introduction

Nowadays, the majority of skin-care products have more than one purpose. Therefore, there is an increasing trend towards advanced materials encapsulating ‘all-in-one’ properties, with evolving products capable of cleaning moisturizing and nourishing the skin simultaneously, to save the consumers' time and money [1–4]. To achieve this, typically, moisturizing active agents and skin nutrients are integrated in the skin-cleaning formulations, leading to products that remove grease and dirt from the skin while at the same time depositing all the beneficial ingredients, thus providing the skin with a ‘healthy finish’ and long-term protection from dehydration and ageing [2,4,5]. Therefore, to continuously develop improved skin-care products while integrating multiple active components and understanding the underlying mechanisms aimed at improving the deposition and uptake of active agents on the skin, it is crucial to design and develop an improved skin mimic for the consistent study and characterization of the retention process. Exploitation of the optimized design principles found in biological systems is highly desirable for synthetic biomimics. If successful, such sophisticated surfaces would enable a new platform for a variety of applications, ranging from smart surfaces, through cosmetics to robotics and biomedicine.

The surface free energy (SFE) of biomimics is often used as a representative parameter of a successful skin replica with the interactions between a solid surface and a liquid substance being highly dependent on the SFE of the solid and the surface tension of the liquid [6–8]. Typically, SFE is calculated indirectly, via measuring the contact angle (CA), using the sessile drop technique, which is based on principles described by Fowkes [7], according to which the total SFE of a solid material can be divided into independent energy components, with each one related to the specific contributing interactions:1.1
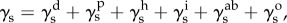
where 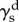 is the dispersive, 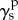 is the polar, 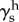 is related to the hydrogen bonds, 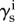 is the induction, 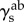 is the acid–base components and 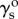 corresponds to all the remaining interactions. Further, Owens & Wendt [8] demonstrated that all the energy interactions which include the SFE, while excluding the 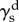, are associated with the polar component, and therefore the SFE of a solid material can be described as the sum of polar and dispersive components:1.2
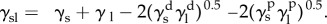
Substituting Young's equation, [6–11]1.3
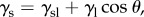
into equation (1.2) yields a correlation of CA to the SFE components of a solid surface:1.4
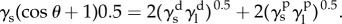


Equation (1.4) together with surface tension values of known liquids and contact angle measurements of these can be used to calculate the total SFE of a solid and its polar and dispersive components. Furthermore, when the polar components of one of the liquids is negligible, equation (1.4) can be simplified as1.5
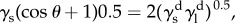
allowing the calculation of the dispersive component of the solid and the liquid. Thomsen [12] have subsequently applied Fowkes theory while performing sessile drop experiments using a KRUSS drop shape analysis to measure the CA and calculate the SFE of skin, obtaining values of 43.7 and 32.9 mJ m^−2^ for non-degreased and degreased skin, respectively; and Krawczyk [13] used the Van-oss approach to calculate SFE values of 44.78 ± 0.66 mJ m^−2^ for non-degreased and 36.05 ± 1.18 mJ m^−2^ for degreased skin. Typically, representative SFE values for human skin are considered to be in the range of 30–45 mJ m^−2^, depending on the body part, ethnicity, sex, age and type of the skin [13].

To improve retention of active skin-care products, an optimal skin mimic is essential to simulate the representative characteristics found on the stratum corneum of human skin. Among the representative properties of mammalian skin, including SFE, charge and reactivity, texture morphology is one of the most important features, because product deposition and coverage can be determined if the keratinous tissues are replicated accurately. Considerable research efforts have been thus focused on studying and developing human skin replicas using a range of polymers [14–17]. However, the vast majority of the earlier fabrication routes to generate a skin mimic with suitable reproducibility and compatibility were cumbersome and based on multistep processes, combined with the subsequent coating of the mimics with monomers to tune the SFE [17], thus compromising the topographic accuracy and altering the physical properties of the roughness and porosity of the synthetic surfaces and entrapment of materials. Facq [14] pioneered the resin replication technique, followed by studies optimizing this method for various applications. The mimics exhibited similar SFE values to real skin i.e. 32 ± 1.0 mJ m^−2^, as well as high control of dimensions and thickness. Datta [17] and Goldman *et al*. [16] have further used alginates and silicon rubber, respectively, for the negative and polyurethanes for the positive replicas, achieving SFE values of 35–45 mJ m^−2^. Charkoudian [15] introduced a model skin surface comprised of gelatin, a synthetic lipid substance and water. By tuning the lipid-to-protein ratio, the generated replica exhibited an SFE of 33.4 mJ m^−2^ with controllable water content. Although progress has been made in mimicking skin by using various fabrication routes including soft imprinting, mechanical etching [17] and micro-moulding, [14–16], only some of the benchmark properties of natural skin, e.g. multilevel micro- and nano-topographic features, hardness and SFE have been achieved to date with no optimal replica of human skin [14–17].

Cosmetic product formulations have been, concurrently, a topic of extensive research and applications [1–3,18–26]. However, none of these studies have paid attention to the characterization and understanding of retention of the hydrophobic i.e. conditioning active agents from cosmetic emulsions on human skin as well as the optimization of skin biomimics for such purposes. Here, we demonstrate the fabrication of enhanced, reproducible biomimetic hierarchical skin replicas based on an optimized version of the polymer resin replication technique, which closely mimics human skin characteristics, combining the topographic structure, SFE and its individual components and mechanical properties. We have also synthesized a representative cosmetic emulsion as a model system to study the retention processes on the developed skin mimics, consisting of a water phase, stabilizing fatty acids and petrolatum, which is considered one of the most effective moisturizing active agents in the cosmetic industry [20,27–31], creating an occlusive layer, sealing the skin and preventing trans-epidermal water loss, thus hydrating the human keratinous tissue in the stratum corneum [20,29,30–33].

Furthermore, a dedicated, innovative set-up was designed and engineered for the accurate deposition of the model system formulation on the novel skin mimic with a controlled lateral force, number of cycles and speed of spreading. The dependence of petrolatum particle size and shear is found to play an important role in the retention processes, thus highlighting the importance of structural parameters, previously overlooked in designing and fabricating skin mimics that combine topographic accuracy and representative surface energy as well as eliminating multiple step errors and the need of extra material coatings to achieve these properties. The robustness and reproducibility of the mimics can be attributed to the design, engineering and precise topographic replication process combined with the correct choice and preparation of materials, yielding an enhanced conformal replica of the skin's surface elements.

## 2. Results and discussion

As an initial step, the fabrication of a robust, simple-to-make, yet accurate skin mimic exhibiting the representative parameters, including the topographic features, SFE and the mechanical properties, was essential. The process commenced with careful preparation of the area of skin to be replicated (live human subject, forearm, Caucasian male, 35–45 years old) followed by heating the positive and negative mimic polymers to 28°C to achieve lower viscosity and allow more effective mixing of the parts. Degassing post mixing was a crucial key factor in achieving improved topographic characteristics while eliminating any trapped bubbles, which can compromise the accuracy of the biomimics. A skin-friendly, medical grade, non-platinum-based polydimethylsiloxane (PDMS) was then directly applied onto the pre-cleaned skin, cured at room temperature and carefully removed. Subsequently, the positive mimics were fabricated in a low-pressure environment, achieving enhanced coverage between the negative and the positive mimic materials.

 As a representative range of hardness for human skin, of various types and complexions, is typically considered between 20 and 40 Shore A [34,35], polyurethanes that displayed hardness values from 10 to 30 Shore A were selected for the positive replicas, aiming to achieve an accurate representation of the force distribution during the deposition step of the product onto the skin mimic. The four optimal polyurethanes chosen for the fabrication were Vytaflex 10 (PU1), Vytaflex 20 (PU2), Poly 74–29 (PU3) and Poly-flex (PU 4) (table 1).

**Table 1.** Mechanical properties of the chosen polymers for fabrication of the skin biomimics.

	PU1	PU2	PU3	PU4
hardness (Shore A)	10	20	30	20
tensile strength (PSI/MPa)	520/3.59	250/1.72	200/1.38	200/1.38

**Figure 1.**
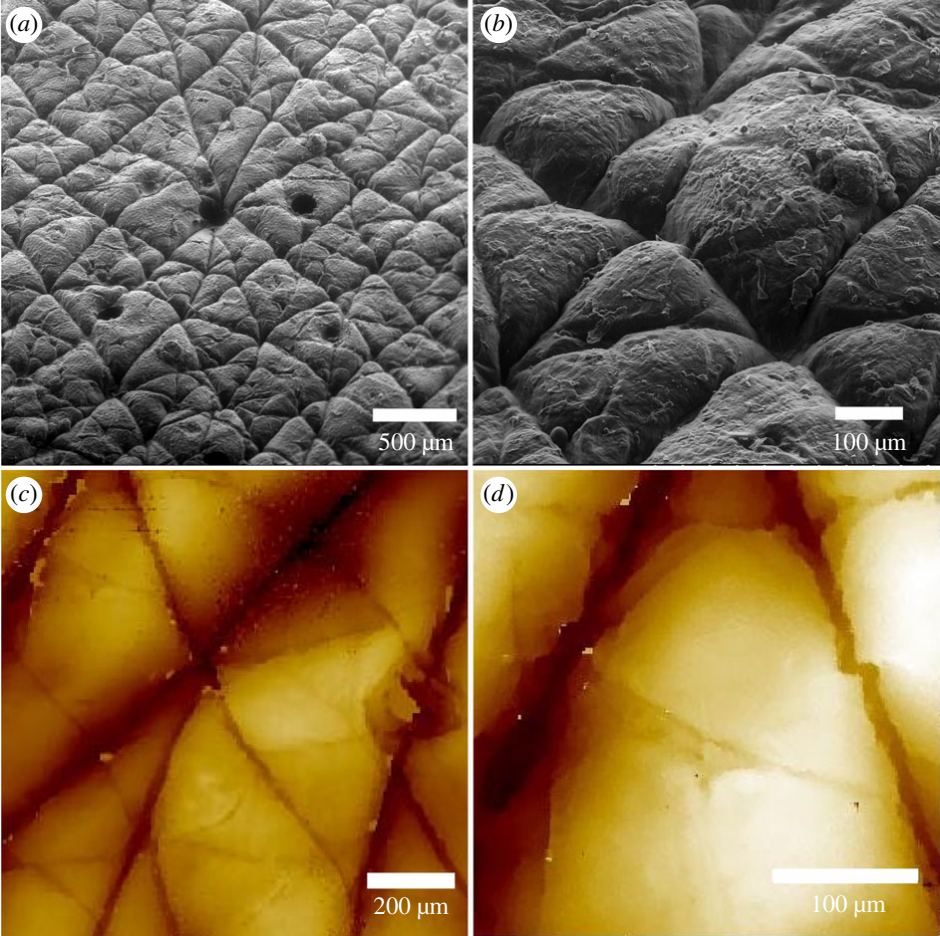
A large area (*a*) and a zoomed-in (*b*) SEM micrograph of the PU4 biomimic showing the high-fidelity replication of the human skin with 3D topography and pore diameters. Representative two-dimensional interferometry overview (*c*) and zoomed-in (*d*) images showing the high-accuracy replication of the skin's mimic topography. (Online version in colour.)

The topographical features and the average roughness of the skin mimics were subsequently characterized using scanning electron microscopy (SEM) and interferometry (figure 1). To determine the distribution of pore sites, we performed quantitative analysis of the SEM and interferometry images over the entire image area (*n* = 5) (figure 1*a–d*), revealing an average diameter of 84.70 ± 0.57 µm. The mean diameter of the pores i.e. of the openings of the stratum corneum connected to the sweat glands, can vary depending on various parameters such as age, ethnicity, sex and skin pore characteristics, typically exhibiting a range of diameters from 60 to 80 µm and a distribution of 200 pores cm^−2^ [36–38]. The SEM images (figure 1*a*,*b*) demonstrate high-replication accuracy of the fabricated skin mimics exhibiting three-dimensional topography and pore distribution, closely similar to human skin, i.e. 40–80 µm in diameter and a distribution of 200–300 cm^−2^ [36], thus outperforming the replication efforts previously found in the literature [14–17]. The high level of precision can be attributed to both the correct choice of materials and the fabrication process itself (see Material and methods). The materials used for the replicas need to, crucially, exhibit low viscosities and free flow to ensure an effective coverage of the air pockets on the surface. The materials used in our fabrication process were heated to 25–30°C to enhance flow and coverage. Additionally, during the positive mimic fabrication, the whole system was placed in a vacuum to enable effective removal of any trapped air between the two materials. The low shrinkage during the curing process enabled mimics to remain accurate and reusable after long periods of time without compromising their quality. An additional benefit of the developed method was the small number of steps required for the production of the mimics, which resulted in reduced cost, time and experimental errors. Interferometry measurements further provided information on the mimics’ roughness (figure 1*c*,*d*) with the skin mimic exhibiting a roughness of 15.48 ± 0.33 µm, comparable to the average values of 10–30 µm reported in the literature [16,17,36,39].

The SFE of the fabricated skin mimics was subsequently calculated using Fowkes theory, which included the CA measurements of diiodomethane and water (table 2). While all four of the fabricated mimics exhibit values comparable to those found in the literature for human skin, depending on the grease concentration of the surface, PU4 appears to have the closest dispersive component to both degreased and non-degreased skin along with the highest polar component among the materials tested, and, therefore it was chosen for the further retention experiments as an optimal replica.

**Table 2.** Characteristics of polymer skin mimics and the human skin.

	PU1	PU2	PU3	PU4	human skin [13]
surface energy (mJ cm^−2^)	40.96 ± 0.07	34.89 ± 0.06	41.51 ± 0.05	38.06 ± 0.51	36.05 ± 1.18
dispersive component	40.60 ± 0.00	34.55 ± 0.00	41.49 ± 0.00	37.68 ± 0.00	34.09 ± 1.47
polar component	0.36 ± 0.07	0.25 ± 0.06	0.02 ± 0.05	0.38 ± 0.51	1.96 ± 0.48

**Figure 2.**
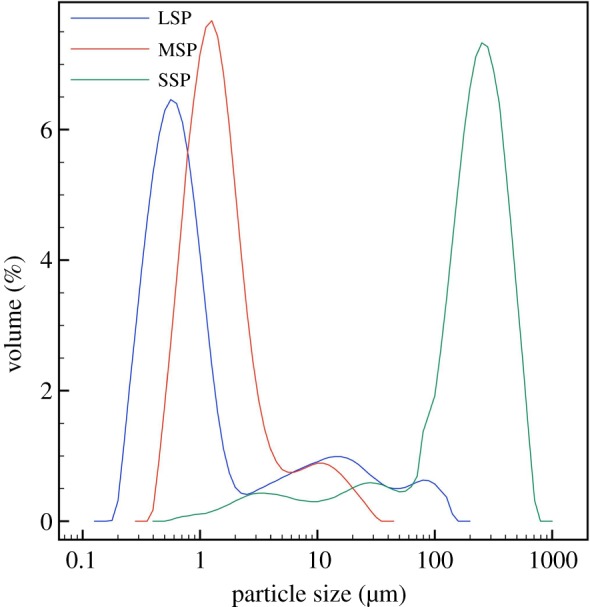
Particle size distribution in (*a*) low-shear (1000 r.p.m.), (*b*) medium-shear (2500 r.p.m.) and (*c*) high-shear (4000 r.p.m.) conditions, yielding the LSP, MSP and SSP emulsions, accordingly indicating that applying higher energy to the formulation step by increasing the rotational speed in the high-shear homogenizer results in emulsions with smaller particle sizes. (Online version in colour.)

A specific, industry-guided emulsion, incorporating only the paramount ingredients for creating a base which can be used in the formulation of a final cosmetic product, was developed for our studies of the retention process and consisted of 20%w/v sodium dodecylbenzenesulfonate, as an anionic surfactant, 20%w/v lauric acid, 20%w/v petrolatum and water. Particle size distribution in the emulsion was studied using Mastersizer with a range of shear applied to the system during the mixing step (figure 2, table 3). In the low-shear emulsion, the largest fraction of particles was found to be in the range of 100–500 µm in diameter, with nearly 75.0% exhibiting diameters larger than 100 µm, producing a relatively uniform emulsion (figure 2*a*). The medium shear emulsion exhibited high uniformity and smaller particle dimensions than those of the low shear emulsion with 67.7% of the particles exhibiting a diameter between 1 and 10 µm and 26.2% of the particles with nanoscale dimensions (figure 2*b*). The high-shear emulsion yielded nano-emulsion properties, displaying the smallest dimensions of particles compared to the other two, with 61.8% of the particles found to be below 1 µm (figure 2*c*). Furthermore, this emulsion was of a low uniformity, possibly due to the potential breakdown of structure under the increased stress during the mixing.

**Table 3.** Distribution of particle size ranges for the low-, medium- and high-shear emulsions.

	particle diameter (µm)				
	0.1–1	1–10	10–100	100–500	500–800
low-shear emulsion (volume%)	0.43	6.42	11.7	88.20	4.1
medium-shear emulsion (volume%)	26.17	67.67	6.21	0	0
high-shear emulsion (volume%)	61.83	21.91	14.75	1.52	0

**Figure 3.**
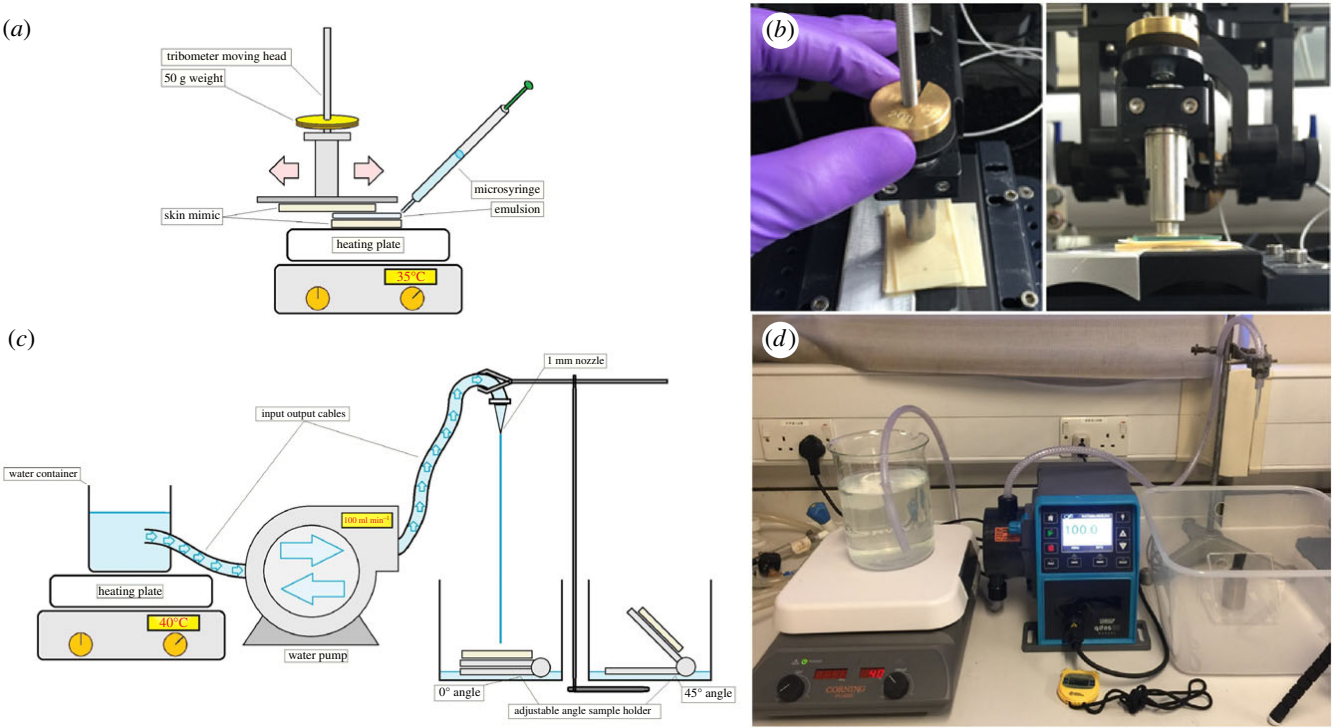
(*a*) Schematic representation and (*b*) the corresponding photograph of the engineered deposition set-up with controllable experimental parameters. The upper moving part of the tribometer was modified to enable a skin mimic-gel-skin mimic geometry. A heating plate was used to maintain skin-like temperature, and the standard normal load of 50 g was applied during the deposition step. Schematics (*c*) and a corresponding photograph (*d*) of the dedicated cleaning set-up used in the retention experiments. Water was heated to 40°C and subsequently delivered through a 1 mm nozzle directly onto the skin mimic at an accurately controlled water pump flow rate. (Online version in colour.)

To establish a controlled and repeatable deposition process of the emulsions on the skin biomimics, we have designed and constructed a dedicated rig combined with a modified tribometer set-up (figure 3*a*,*b*). The skin mimic-to-skin mimic interface was accomplished and controlled with the bottom mimic maintained at a temperature similar to that of human skin. Emulsion (1 ml) was then deposited between the two mimics at a controlled speed of 10 mm s^−1^ and five cycles. (Figure 3*a*,*b*) Subsequently, a dedicated cleaning set-up was designed and engineered as a scaled-down version of a shower head.

However, instead of the multiple jets typically found in the shower, a single water jet set-up was developed by combining a high-accuracy water pump, tubes, 1 mm ending nozzle, a water tank to feed the pump, a heating plate to regulate temperature, a thermometer and a sample stage to hold the mimic at the desired angle (figure 3*c*,*d*). The working conditions of the pump were calculated in accordance with the UK and US regulations and consumer surveys. The optimal skin biomimics were then systematically exploited for studying the retention process of petrolatum with the developed emulsion formulation. Briefly, 1 × 1 cm^2^ skin mimics were initially weighed, followed by the deposition of 2 ml of emulsion using a standard lateral load of 100 g and the tribometer set-up, and, finally, weighed again and moved to the cleaning apparatus where the mimic–emulsion interface was cleaned using a water jet set-up for 30 s at 90° flow and, subsequently, dried in ambient conditions, weighed and characterized using fluorescent microscopy. A lipophilic, non-soluble fluorescent dye was used to stain the oily phase during the emulsion fabrication step and thus enable retention to be monitored via fluorescent microscopy.

**Figure 4.**
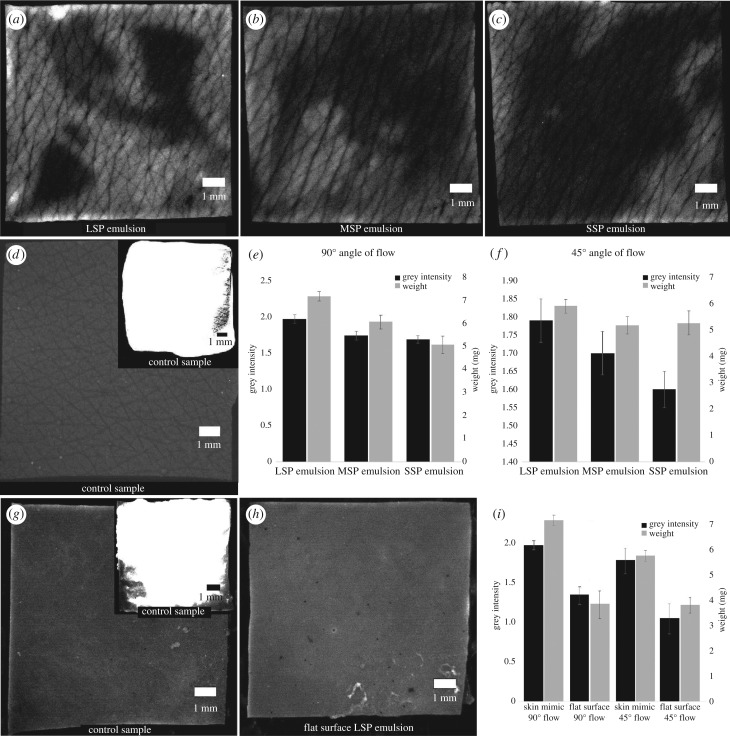
Retention analysis of the fluorescent images of the (*a*) LSP, (*b*), MSP and (*c*) SSP emulsions in comparison to the control samples (*d*) of the clean skin mimic with no product deposited and (inset) the skin mimic after the product deposition step, where it is covered with a layer of product. Intensity and retention, weight analysis of the retained product amounts as a function of the emulsion particles size distribution at (*e*) 90° and (*f*) 45° flow. (*g*) A clean flat sample, with no product deposited with the post LSP emulsion deposition step shown in the inset and the (*h*) flat sample after the cleaning step. (*i*) Retention analysis via the intensity and weight distribution of the retained emulsion amounts on flat samples versus skin mimics at 90° and 45°.

Figure 4*a*–*c* demonstrates the retention levels of large, medium and small particle size emulsions on mimics at 90° of flow. The large size particle (LSP) emulsion yields the largest coverage of petrolatum in comparison to the medium size particle (MSP) and small size particle (SSP) emulsions, with the latter exhibiting very low retention levels. The brighter areas represent areas with higher fluorescent signal, which in turn indicate the presence of a petrolatum film. Owing to the greasy nature of petrolatum, this layer adheres to the skin and is hard to remove, thus diminishing the trans-epidermal water loss, trapping the moisture in the outer layers of the skin. This is in agreement with petrolatum hydration mechanisms, [20] showing that when petrolatum is applied on the skin, the combination of shear and body heat softens the latter and creates an occlusive layer on the stratum corneum.

The retention levels were also characterized using Matlab-based image analysis of the fluorescent microscopy images (figure 4*e*,*f*) by calculating the intensity levels of the fluorescent signal. The LSP emulsion was found to exhibit maximal retention at both 45° and 90° of flow with the highest intensity signal. Under vertical flow, the LSP emulsion showed an average relative intensity value of 1.97 ± 0.06, whereas the MSP emulsion showed a value of 1.74 ± 0.06 and the SSP emulsion yielded 1.69 ± 0.05. Nevertheless, at 45° flow, the LSP emulsion exhibited relative intensity levels of 1.79 ± 0.06, the MSP of 1.70 ± 0.06 and the SSP showed 1.60 ± 0.05 average intensity. These results indicate that the presence of larger particles leads to improved retention of petrolatum on the skin and that a tilted angle of flow i.e. at 45° yields considerably more intensive removal of the deposited emulsions. Particularly, the LSP samples that were cleaned at 45°, exhibited lower retention by up to 18.00 ± 0.36% in comparison to those cleaned via vertical flow, while the MSP emulsions demonstrated a reduction of 4.00 ± 0.31% in retention at 45°, with the SSP yielding a similar decrease of 9.00 ± 0.37%, when cleaned at a tilted angle.

The weight of the retained emulsion was also studied via weight measurements and gravimetric analysis. Herein, the remaining portion of the emulsion was calculated by subtracting the initial skin-mimic weight value from the post-cleaning value, yielding a complementary retention parameter. The LSP emulsion revealed a higher degree of retention i.e. an increase of 12.27 ± 1.34% in comparison to the MSP and 27.73 ± 0.27% higher compared to the SSP emulsions (table 4), which is consistent with the observations from the fluorescent microscopy characterization and image data analysis (figure 4). Furthermore, from both the gravimetric and the grey intensity Matlab analysis, the 45° angle of flow leads to lower values of retention in comparison to the 90°, for all the emulsion formulations (table 4), with LSP emulsion samples resulting in a 21.39 ± 0.42% reduction in retention and the MSP emulsion samples showing a 17.02 ± 0.54% reduction in retention in comparison to the vertical flow cleaning.

**Table 4.** Gravimetric analysis of LSP, MSP and SSP emulsions on 90° and 45° flow.

	weight (mg)					
	LSP	s.d.	MSP	s.d.	SSP	s.d.
90°	6.77	0.06	6.03	0.84	5.30	0.17
45°	5.91	0.26	5.17	0.33	5.27	0.45

Furthermore, the impact of topography on retention was also studied by evaluating the deposition and cleaning process on control samples consisting of flat polyurethane surfaces in comparison to the skin mimics. The LSP emulsion was applied, following the same deposition protocol onto the flat PU1 surfaces with flow rates at 90° and 45°. The flat surfaces exhibited reduced retention (figure 4*g*–*i*) with the relative intensity, at vertical flow, found to be 1.35 ± 0.27, which is a significant 31.47% reduction in comparison to the equivalent skin mimic. At 45° cleaning, the relative grey intensity value was reduced to 1.07 ± 0.27, which is a 40.22% reduction in comparison to the skin mimic. Gravimetric analysis further corroborated the grey intensity data (figure 4*i*), with flat surfaces cleaned with a vertical flow exhibiting residues of 3.92 ± 0.25 mg cm^−2^ and those cleaned at 45° showing product retention of 3.87 ± 0.17 mg cm^−2^. Thus, a significant reduction in retention in comparison to the skin mimics was observed with an overall 42.10% at 90° flow and 34.52% at 45° flow, which is attributed to the elimination of physical entrapment phenomena. Noticeably, both flat and skin-mimic samples, at 45° flow during the cleaning process, lead to lower retention levels.

**Figure 5.**
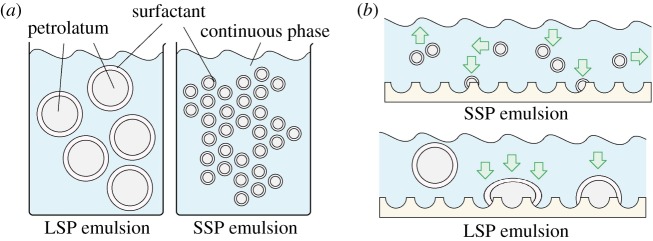
Schematic representation of: (*a*) LSP (i) and SSP (ii) emulsion demonstrating the noticeable difference in interfacial surface area which leads to the counter effect of surfactant in the case of SSP and the enhanced retention in the case of the LSP emulsions. (*b*) Skin mimics with an applied layer of SSP and LSP emulsions: for the LSP emulsion, the considerable effects of gravity and shear forces lead to the particles' deformation and break-up and, thus, higher retention values. By contrast, in the SSP emulsions, the particles are more stable and resistant to thermal and mechanical stresses, and exhibit high mobility and enhanced Brownian diffusion forces, all enabling an ease of removal during the cleansing process, without adhering or releasing oils onto the skin mimic's topography. (Online version in colour.)

Three possible mechanisms can lie at the origin of the improved retention observed in our LSP formulations in comparison to the MSP and SSP emulsions: (1) lower surface area effect reducing the impact of surfactant, (2) improved oil particle packing due to the enhanced polydispersity of particles' distribution in LPS emulsions and (3) lower mobility of larger particles combined with the deformation and deconstruction of the emulsion. It is well established that the surfactants that are embodied in a cosmetic formulation serve multiple purposes with the major purpose of cleansing the skin of the undesired substances such us dirt, sebum and excessive oil along with acting as emulsifiers to stabilize the oil particles inside the product. However, surfactants are also known to exhibit a counter effect during the deposition and the retention processes of the desired oils onto the skin [40–42]. As the surfactants cannot distinguish between beneficial and the redundant oils, a considerable proportion of the materials aimed for retention purposes remain trapped in a layer of surfactant and, subsequently, are washed away during the cleaning step. To experimentally address the question, we have found that the LSP emulsion yielded improved retention, which is attributed to the dimensions of the particles of the emulsion affecting both the deposition and retention steps. This can be explained by the fact that the SSP emulsion has a considerably higher surface area in comparison to the LSP emulsion (figure 5*a*).

We have further calculated the interfacial surface area of the emulsions while making the assumptions that the oil particles are spherical in nature and the average diameter of the majority of particles in each emulsion is equal to the median, according to the Mastersizer data [43]. For the LSP emulsion, the median particle diameter is found to be 251.54 µm and the calculated surface area for 1 ml of emulsion, which is the amount applied to a single skin mimic, is 0.024 m^2^. In the case of the MSP emulsion, the median particle diameter is 1.41 µm and the calculated surface area is 6.24 m^2^. The SSP emulsion has the smallest median particle diameter of 0.71 µm and, in contrast, the calculated surface area is the largest, reaching 8.45 m^2^. These results indicate that the surface area in smaller particle size emulsions is considerably higher than in large particle size emulsions and because this interfacial area is covered by a film of surfactant, the impact of the above-discussed counter effect of surfactants is considerably higher in the SSP emulsions [44].

The second mechanism is related to the higher level of polydispersity of particle sizes in particular, in the LPS emulsions (figure 2). This extended range of particle sizes can lead to improved packing of the oil droplets inside the topographic features of the skin mimic. Improved packing can promote minimal interfacial forces between the oil droplets in oil-in-water emulsions, leading to improved structural and mechanical properties throughout their volume [45]. Additionally, it has been demonstrated that monodisperse sphere systems exhibit limited maximum packing fractions of *φ*_f_ = 0.64, regardless of particle size. In polydisperse systems the maximum packing fraction values increase due to the smaller particles filling in the voids between the larger ones, consequently yielding mechanically stronger structures. For the bimodal systems, the maximum possible packing fraction was found to be *φ*_f_ = 0.87. The size ratio of particles in emulsions greatly influences their properties, with those exhibiting a broad range of particle dimensions leading to improved packing abilities [46,47]. In our study, all the emulsions are bimodal and, therefore, demonstrate a certain level of improved packing. The LSP emulsions exhibit the largest distribution of particle sizes and, therefore, are anticipated to have optimal packing abilities. During the deposition step, where a given shear is applied onto the product and the mimic, the LSP emulsions are expected to yield an improved physical entrapment of the oil particles inside the skin mimics’ topography, with the smaller particles to fill in the smaller ridges and gaps, and the larger ones, the bigger pores and skin discontinuities. However, the particles in our study require a degree of deconstruction in order to release their content onto the skin's topography and although enhanced packing will result in a higher concentration of oil particles inside the mimics' topographic features, the energy required to break those particles is considerably higher for the effective release and deposition of these hydrophobic substances, rendering this mechanism less plausible.

Furthermore, it is useful to estimate the potential role of particle deformation in improved retention. To assist the deposition and retention of the oily phase on the surface of skin mimics, the oily particles must be reorganized and broken to enable the release of the dispersed material. Smaller particles in oil-in-water emulsions are known to exhibit enhanced stability and resistance to shear stress and temperature in comparison to the larger ones [43,44,48]. Subsequently, because less energy is required to release the oil from the larger particles on the surface of the mimic, smaller particle emulsions result in lower retention levels (figure 5*b*). Iyer & Cayatte [48] have demonstrated that oil-in-water emulsions of smaller size particles are more stable under heat and shear variations relative to the larger size particle emulsions. Tjarwat [43,49,50] have presented the superiority in stability and energy required to demulsify smaller particles, referring to the effect of gravity on the LSPs within emulsions, and have shown that submicron particles demonstrate Brownian diffusion with *k*T values that surpass the gravitational forces and, thus, the SSP emulsions are energetically more stable and do not easily phase separate in comparison to the LSP emulsions. The correlation of particle size and the effect of gravity forces can be estimated using2.1
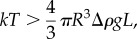
where *k* is the Boltzmann constant, *T* is the temperature, *R* is the particle radius, Δ*ρ* is the density difference between oil and medium, *g* is gravity and *L* is the height of the vessel. For the LSP and the SSP emulsions, the gravitational forces are calculated using the following equations:2.2
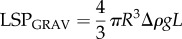
and2.3
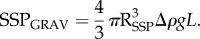


Making an assumption that the *R*_LSP_ and the *R*_SSP_ are the median particle size diameters, both kept in a vessel of the same height and that all the other components of equation (2.3) are identical for both emulsions enables calculating the forces, which are only affected by the particle diameter in each emulsion, yielding LSP_GRAV_ > SSP_GRAV_ ⇔ 251.54^3^ *>* 0.71^3^ ⇔ 1.59 *×* 10^7^ > 0.36. Therefore, the calculated gravitational impact is found to be 4.44 × 10^7^ times higher in the LSP in comparison to the SSP emulsions. Therefore, larger particle dimensions greatly increase the impact of gravity forces on the oil particles of the emulsion and if those become greater than the Brownian diffusion forces, phase separation and destabilization of the emulsion are more likely to occur. The effect of gravity on large particles renders them considerably less mobile, which, combined with the fact that they are more prone to shear stress and deformation than smaller particles, results in enhanced deposition of the oil onto the skin mimic, with less energy required for their deformation or break-up. Thus, the LSP emulsions lead to increased values of retention, whereas the smaller particles are considerably less affected by gravitational forces and are easier to wash away due to their high mobility and resistance to shear stress. Both, the energetically favourable deformation of the LSP emulsions and the influence of the interfacial surface area on the surfactant counter effect suggest that these two mechanisms may well be dominant in our experiments. The detailed interplay of these two effects and the microstructure of the emulsions will be the subject of further studies.

## 3. Conclusion

In this study, a dedicated system has been designed and engineered to mimic the fundamental principles describing the typical showering procedure and the skin-to-product interactions. An advanced and improved skin mimic was fabricated and used as a working sample base with a synthesized model emulsion formulation, followed by the development of a novel rig for product deposition and, consequently, systematic retention studies were performed. The developed mimics and set-ups enabled a cost-effective, controlled and highly reproducible approach to study the impact of different physical properties of the formulations and working parameters, thus bridging the gap between the retention of active agents on human skin and the associated characterization methods. The fabricated skin-mimic exhibited improved characterizes including, precise skin-like topographic features, accurate mechanical properties and surface energy, all closely mimicking real human skin. The developed deposition set-up enabled increased control of parameters. The optimized water jet in the cleaning set-up was found to exhibit an improved control of the flow rate, tunable flow angle and the water temperature while accurately representing the in-shower conditions.

Furthermore, the impact of petrolatum particle sizes and angles of flow on the retention have been studied with LSP emulsions exhibiting higher levels of retention at both flow angles. This was attributed to the microstructure of the emulsion and the high internal concentration of surfactant. The improved retention was found to predominantly arise from the emulsion's intrinsic particle size, which, kinetically and in terms of the surface energy, enhances the retention of the formulation. Our results provide a model study on generic cosmetic emulsions, which with the improved retention properties, may pave the way for the technological or scientific relevance in, for instance, skin moisturization, repair and protection as well as anti-ageing and anti-wrinkle products. Furthermore, design, fabrication and control of functional advanced skin biomimics with controllable micro-patterned morphology and properties may also have potential applications in the pharmaceutical and cosmetic industries.

## 4. Material and methods

### 4.1. Materials

Skin-safe silicone PDMS (Body-double Kkinsafe Si), alginate (alja-safe and alja-safe breeze liquid alginate) and polyurethanes VytaFlex^®^ 10 and VytaFlex^®^ 20 were purchased from Bentley Advanced Materials. Polytek Poly74–29 Flexible Polyurethane Rubber and Polytek Poly PT Flex 20 Liquid Casting Rubber were purchased from MB Fibreglass. Diiodomethane, ethylene glycol and water of HPLC grade were purchased from Sigma-Aldrich. Sodium dodecyl sulfate, sodium dodecylbenzenesulfonate, cationic surfactants-WP, lauric acid, vaseline, cetyl-alcohol and difluoro{2-[1-(3,5-dimethyl-2 h-pyrrol-2H-pyrrol-2-ylidene-N)ethyl]-3,5-dimethyl-1H-pyrrolato-N}boron Pyrromethene546 were purchased from Sigma-Aldrich, Polysorbate80 was purchased from EPS-Engineering.

### 4.2. Skin-mimic fabrication

Initially, the skin area of interest was identified and prepared for the replication. The chosen area was 5 × 5 cm^2^, human forearm (from live male subjects) and the preparation process included shaving the area with a razor, cleaning the area with soap and water and rinsing off for 2 min. Subsequently, two parts of the PDMS (specific gravity: 1.17 g cm^−3^, viscosity: 5000 mPa·s) were combined, achieving a homogeneous mixture which was then de-gassed under vacuum for 5 min, followed by an application of a thin layer of PDMS (3 g) onto the clean skin area and cured at RT in 15 ± 2 min. The PDMS was then detached from the skin and the negative replica was stored in clean-room conditions. For the positive replica, a range of polyurethanes was used (Polytec polypt-flex, Poly74-29, Vytaflex 10 and Vytaflex 20). All the materials were prepared for curing by combining the product parts together, and these were applied on top of the negative mimic and kept under vacuum for 10 min, which enabled efficient removal of trapped air from the fine topographic features of the mimic, leading to a high interfacial contact area between the two materials. The assembly was annealed overnight at 70°C and the final replicas were stored in clean conditions.

### 4.3. Preparation of the model system emulsion

Surfactant (20 g) was mixed with 50 ml of water under continuous heating at 70°C and stirring at 500 rpm. Once a homogeneous mixture was accomplished, 20 g of fatty acids, which were kept at 70°C, in a liquid form, were added to the mixture turning it into a more viscous, gel-like form. Water was then added to the mixture to a total volume of 80 ml. Consequently, 20 g of petrolatum was melted at 45°C and 10 ul of an acetone/pyrromethene solution with a concentration of 0.05% w/w was added to it. After the dye solution was fully dissolved in petrolatum, the mixture was added to the water and both phases were mixed using a high-shear homogenizer. Using the homogenizer at 1000, 2500 and 9000 rpm, low-shear, medium-shear and high-shear emulsions were prepared, accordingly.

### 4.4. Deposition set-up

For controlled and repetitive deposition of the emulsion on the skin mimic, a modified tribometer-based set-up was used. The top, moving part was modified to enable a conformal fit of the skin and, therefore, enabling the skin mimic-to-skin mimic interface, closely representing the real-world system. To incorporate the impact of human skin temperature on the deposition process, the bottom part of the tribometer was removed and replaced with a heating plate, allowing the mimics to be at a temperature of 35°C, similar to human skin *in vivo*. A typical protocol included placing the samples on the heating plate and holding them stable with double-sided tape. Consequently, 2 ml of emulsion was deposited on top of the sample using a syringe and a standard weight, 50 g, was applied on top of the moving part of the set-up and the moving sequence was set to five cycles with a speed of 10 mm s^−1^. Once the deposition procedure had finished, the mimics were carefully removed from the heating plate and placed on the cleaning apparatus.

### 4.5. Cleaning set-up

A downscaled simulation of a shower was designed, in the form of a single water jet set-up. This was achieved using a high-accuracy pump, a heating plate, a water tank, input and output tubes, a nozzle with a 1 mm hole and a variable angle sample base. The working parameters of this set-up were calculated in accordance with the UK and US regulations for shower conditions. Most showerheads consist of an area of multiple water jets adjacent to each other. Typically, the diameter of these water jets is 1 mm and the number ranges from 70 to 100, depending on the make and country of origin. To calculate the flow rate for the single jet, an assumption that showerheads consist of approximately 85 single jets was made and then, by dividing the maximum working flow rates for UK and US households by this number, the calculated flow rate obtained was between 95 and 110 ml min^−1^ and, thus, it was set at 100 ml min^−1^ in our experiments. The water tank was placed on top of the heating plate at 40°C, which is in the range of acceptable shower temperatures according to the NHS. The nozzle was placed 10 cm above the sample and the rinsing time was set at 30 s. For this set of experiments 1 × 1 cm^2^ samples of skin mimics were used. After the cleaning process took place, the mimics were carefully removed from the stage and left to dry in ambient conditions for 10–20 min. The mimics were kept in a plastic container to avoid environmental contamination. The most fundamental parameters that describe the cleaning process during a shower are the flow rate of water coming out of the showerhead, the temperature and the total cleaning time. According to the UK regulations, the maximum flow rate for showerheads is up to 8 l min^−1^ and 5 bar pressure. The US Department of Energy suggests maximum showerhead flow rates of up to 9.5 l min^−1^ and 5.5 bar pressure. According to Health and Safety Executive published by the UK government, the highest allowed temperature in showers is 44°C. According to a consumer habit survey [38], the average shower time is 8 min with the most common shower time, by statistics [51], ranging from 5 to 10 min. Based on these, a flow rate between 8 and 9.5 l min^−1^, a water temperature of 40°C and rinsing times of up to 5 min were considered as representative working parameters for the cleaning set-up in our experiments.

### 4.6. Mastersizer

The mastersizer (Malvern-2000) with the optical bench was used to retrieve a scattering pattern from a field of particles and to calculate particle dimensions [52]. The set-up consisted of a water tank with an overhead mixer which provided a feed to a cell where the laser beam impacted the sample solution. Initially, the water tank was cleaned thoroughly with repeated flashes of 5% solution of DECON, a surfactant specifically made for Malvern instruments and large amounts of de-ionized water. When the software showed a small amount of background noise from the cell, the model system emulsion was diluted in the water tank with overhead blade stirring at standard rotational speed. The laser refractive index and laser absorption values for petrolatum were provided before commencing the measurements.

### 4.7. Scanning electron microscopy

SEM measurements were performed using a Hitachi S3400 SEM with a tungsten hairpin source with a lateral resolution of 5–10 nm. The skin biomimics were cut into 1 × 1 cm^2^ and 3 × 4 cm^2^ pieces and fixed on the SEM stage using a conductive double-sided tape with no sputtering. The SEM was set at 10.0 kV in a variable pressure mode at 70 Pa.

### 4.8. Interferometry

The MicroXAM-1200 3D non-contact interferometer was used for analysing surface morphology, roughness, film thickness and topographic characteristics. White light interferometry was used to generate high-resolution 3D images with a high vertical resolution and accuracy. The MapVUE^®^AE analysis software package was used for the parameter calculations, filtering, imaging analysis and automated report generation. The skin mimics were cut and sputtered with gold to enhance white light reflection.

### 4.9. Contact angle measurements

To establish the surface energy of the mimic, and contact angle of water and diiodomethane, a goniometer set-up was carried out. The mimic was placed on a flat surface with a microscopic camera (Andonstar). Subsequently, a 20 µl drop of liquid (water or diiodomethane) was deposited on the skin mimic using a high-accuracy pipette. ImageJ was used to analyse the captured images of the droplets, yielding contact angle values of the two liquids on the skin mimics, used for the calculation of surface energies.

### 4.10. Fluorescent microscopy

The oily phase was dyed using pyrromethene-546, a fluorescent, lipophilic and water-insoluble dye, which exhibited an absorption peak at 495 nm and an emission peak at 505 nm. This microscope was connected to a single-wavelength LED light source (CoolLED) and a camera (MR285MU_BH, Ximea) with the µManager software. Using ImageJ, the acquired images were adjusted in such a way that only the fluorescent parts of the image were visible. Firstly, a control sample which was completely free of fluorescent material was captured as an 8-bit image. The image was subsequently colour-adjusted until the clean mimic was invisible. This way all the noise from the white light was eliminated and only the detectable signal intensity was derived from fluorescent materials.

### 4.11. Image analysis using Matlab

The images acquired from the fluorescent microscope were analysed using the image processing toolbox (Matlab 2016), and a Matlab code was developed to generate quantitative data of the retained petrolatum layer grey intensity, given that only the petrolatum emits a fluorescent signal and that the dye had not affected any other parts of the emulsion. Comparing these values among different samples, the effect of particle size on retention was studied.

## Data accessibility

All data essential to evaluate and establish the conclusions of this study are present in the paper itself and further available upon request from the authors.
